# A Model of Minor Histocompatibility Antigens in Allogeneic Hematopoietic Cell Transplantation

**DOI:** 10.3389/fimmu.2021.782152

**Published:** 2021-11-18

**Authors:** Paul J. Martin, David M. Levine, Barry E. Storer, Xiuwen Zheng, Deepti Jain, Ben Heavner, Brandon M. Norris, Daniel E. Geraghty, Stephen R. Spellman, Cassie L. Sather, Feinan Wu, John A. Hansen

**Affiliations:** ^1^ Clinical Research Division, Fred Hutchinson Cancer Research Center, Seattle, WA, United States; ^2^ Department of Medicine, University of Washington School of Medicine, Seattle, WA, United States; ^3^ Department of Biostatistics, University of Washington, Seattle, WA, United States; ^4^ Center for International Blood and Marrow Transplant Research, National Marrow Donor Program, Minneapolis, MN, United States; ^5^ Genomics & Bioinformatics Shared Resource, Fred Hutchinson Cancer Research Center, Seattle, WA, United States

**Keywords:** genetic variation, graft-versus-host disease, single nucleotide polymorphisms (SNPs), hematopoietic cell transplantation, minor histocompatibility antigens

## Abstract

Minor histocompatibility antigens (mHAg) composed of peptides presented by HLA molecules can cause immune responses involved in graft-versus-host disease (GVHD) and graft-versus-leukemia effects after allogeneic hematopoietic cell transplantation (HCT). The current study was designed to identify individual graft-versus-host genomic mismatches associated with altered risks of acute or chronic GVHD or relapse after HCT between HLA-genotypically identical siblings. Our results demonstrate that in allogeneic HCT between a pair of HLA-identical siblings, a mHAg manifests as a set of peptides originating from annotated proteins and non-annotated open reading frames, which i) are encoded by a group of highly associated recipient genomic mismatches, ii) bind to HLA allotypes in the recipient, and iii) evoke a donor immune response. Attribution of the immune response and consequent clinical outcomes to individual peptide components within this set will likely differ from patient to patient according to their HLA types.

## Introduction

Acute and chronic GVHD and recurrent or progressive malignancy (i.e., “relapse”) represent major outcomes that determine the success of allogeneic hematopoietic cell transplantation (HCT). Acute and chronic GVHD reflect immune-mediated injury mediated by donor cells in recipient tissues, and to some extent, relapse represents a lack of immune-mediated attack on malignant cells that remain viable in the recipient after the pretransplant conditioning regimen. Donor T cells can recognize both major and minor histocompatibility antigens (mHAgs) in HLA-mismatched recipients, but in HLA-matched sibling recipients, donor T cells recognize only mHAgs (1, 2). Minor histocompatibility antigens originate from source proteins or peptides that are processed through the MHC class I and class II antigen processing pathways for binding to major histocompatibility complex (MHC) molecules and presentation to T cells (1, 2). DNA sequence and structural variation between siblings generates mHAg mismatching between recipients and donors. Amino acid differences resulting from single nucleotide polymorphisms (SNP), insertions and deletions (indels) and other types of variants represent well-recognized mechanisms that generate mHAgs (1–4).

The past decade has seen rapid progress in the use of high throughput methods and computational biology to identify mHAgs in humans. Analysis of HLA-identical sibling pairs has estimated the proportion of nonsynonymous peptides that bind to HLA-class I molecules and the proportion of HLA-class I peptides that are polymorphic (5), and simulations with publicly available data have estimated the numbers of polymorphic peptides with strong binding to HLA-class I molecules in sibling and unrelated donor-recipient pairs (6). Others have identified mHAgs by screening patient-derived T cell clones against a panel of sequenced targets expressing common HLA alleles (7). A proteogenomic approach identified mHAgs expressed by hematopoietic tissues but not by other tissues as potential targets for graft-versus-leukemia effects (8). Although the diversity of peptides bound to class I and II HLA-molecules did not correlate with outcomes after HCT (9), the extent of genome-wide mismatching between siblings has been correlated with the risk of severe acute GVHD (10) and chronic GVHD (11). More sophisticated computational algorithms have been designed to predict the overall *in vivo* alloreactive T cell response (12), but their accuracy and utility have not yet been evaluated, and only limited progress has been made toward implicating individual mHAgs in outcomes after HCT in clinical studies (13).

The current study was designed to identify individual graft-versus-host genomic mismatches associated with altered risks of acute or chronic GVHD or relapse after allogeneic HCT between HLA-identical siblings. We initially focused on genomic variants that could produce peptides predicted to bind HLA-A*02:01, the most frequent MHC allele in our study cohort. Associations observed in HLA-A*02:01-postive recipients but not in HLA-A02-negative recipients in a discovery cohort were tested for replication in an independent cohort of HLA-A*02:01-positive recipients. A separate MHC-agnostic analysis was used to identify recipient mismatch associations without taking HLA restriction into consideration.

## Materials And Methods

### Study Population

All recipient and donor blood samples were collected before HCT according to research protocols approved by the Fred Hutchinson Cancer Research Center (FHCRC) Institutional Review Board (IRB) or the National Marrow Donor Program (NMDP). Project-specific IRB approval was obtained for the use of clinical data and research biospecimens.

The FHCRC cohort included 1868 HLA-A, B, C, DRB1, DQA1, DQB1, DPA1, DPB1-matched donor-recipient sibling pairs with mostly European ancestry (~83%) who had a first allogeneic HCT with marrow or growth factor-mobilized blood cells at the FHCRC and Seattle Cancer Care Alliance from 1990 through 2011. Recipients treated with T-cell depleting antibodies or high-dose cyclophosphamide after HCT and those with syngeneic or cord blood donors were excluded. A single prior autologous HCT was allowed. Conditioning regimens were categorized as myeloablative or nonmyeloablative according to the intensity of chemotherapy and total body irradiation. Indications for HCT included hematological malignancy or myelodysplasia. Donors and recipient 4-digit typing of HLA-A, B, C, DRB1, DQB1, DPA1 and DPB1 alleles was determined as described previously (10). We used a cohort from the Center for International Blood and Marrow Transplant Research (CIBMTR), a research collaboration between the NMDP and the Medical College of Wisconsin to test replication of HLA-A*02:01 mHAg GWAS associations discovered in the FHCRC cohort. The CIBMTR cohort consisted of 838 HLA-A, B, C, DRB1, DQB1-matched sibling pairs with self-identified European ancestry and at least 1 HLA-A*02:01 allele. Patients in this cohort had HCT from 2008 through 2018.

### Sample Preparation, Genotyping, Imputation and QA/QC

Details regarding preparation of genomic DNA samples from donors and recipients for the FHCRC cohort and the related DNA amplification, genotyping platforms, hybridization, genotyping, imputation algorithms, quality control and quality assurance have been described previously (10). Targeted sequencing of variants selected for replication using the CIMBTR sample pairs was done by the Genomics & Bioinformatics shared resource at FHCRC using the AmpliSeq PCR workflow on the MiSeq platform (Illumina). Joint genotyping was done using the GATK pipeline 4.1.8.1 following the best practice workflow (14–16). Briefly, picard 2.18 (17) was used to process Fastq and Bam files to generate analysis-ready reads. BWA 0.7 (18) was used to map paired-end reads to reference genome hg19, and GATK 4.1.8 was used to call variants for each sample (BaseRecalibrator, ApplyBQSR and HaplotypeCaller in GVCF mode) followed by joint-calling (GenomicsDBImport and GenotypeGVCFs). Quality assurance, quality control and variant filtering followed recommendations for targeted sequencing from the GATK website (19–21). We further removed variants with high allelic ratios, ancestry outlier samples identified by clustering in the space of the first two principal components, male samples with high X chromosome heterozygosity and sample pairs whose observed identity-by-descent relationship did not match the expected full-sibling relationship. Whenever possible, HLA-A*02:01 replication testing with CIBMTR samples was done with 2 different variants representing each discovery in case the primary assay failed.

### Statistical Analysis

The 6 outcomes tested were acute GVHD categorized as peak grade 2-4, 2b-4 and 3-4 severity (22, 23), stage 2-4 gut GVHD, chronic GVHD and recurrent or progressive malignancy (i.e., relapse). Grade 2b-4 acute GVHD excludes isolated stage 1 gut GVHD, which is frequently recognized at FHCRC (24). Chronic GVHD was diagnosed according to historical criteria (25) because evaluation according to NIH criteria was not available for many patients.

Variants were analyzed in 2 categories according to whether they are predicted to encode differences in the amino acid sequence of an annotated protein in the recipient when compared to the donor, hereafter termed “coding” variants, or not (hereafter termed “noncoding” variants). The coding category included variants whose alleles were predicted to have missense, inframe insertion or deletion, frameshift, start lost, stop gained or lost, stop retained, incomplete terminal codon, or protein altering effect on the overlapping transcript as predicted by the Ensembl Variant Effect Predictor (VEP) (26). The annotations were extracted from the Annotation Explorer application hosted on Biodata Catalyst (https://biodatacatalyst.nhlbi.nih.gov/) (27).

For each variant, recipient mismatching was evaluated separately for the major allele and the minor allele. For a variant with major and minor alleles “a” and “b”, pairs with “bb” donors and “ab” or “aa” recipients were categorized as mismatched for the major allele, and pairs with “aa” donors and “ab” or “bb” recipients were excluded to avoid confounding by mismatching for the minor allele. Similarly, pairs with “aa” donors and “ab” or “bb” recipients were categorized as mismatched for the minor allele, and pairs with “bb” donors and “aa” or “ab” recipients were excluded to avoid confounding by mismatching for the major allele. Recipient allele mismatch associations (RAMAs) for each outcome were based on cause-specific hazard ratio analysis using Cox regression comparing pairs with recipient mismatching versus those without mismatching for a given major or minor allele, treating death as a competing risk for all endpoints and relapse as a competing risk for acute and chronic GVHD.

In the discovery phase of the HLA-A*02:01 analysis, RAMAs were tested by proportional hazards analysis in the FHCRC cohort using 824 HLA-A*02:01-positive donor-recipient pairs with at least 1 HLA-A*02:01 allele and in 929 HLA-A02 supertype-negative donor-recipient pairs who did not have HLA-A*02:01 or other HLA-A02 supertype alleles (A*02:XX, 68:02, 68:15, 68:57, 68:28 or 69:01) (28). Coding RAMAs with likelihood ratio p-values ≤.01 in HLA-A*02:01-positive pairs and p-values ≤.01 for A*02:01 interaction in a combined HLA-A*02:01-positive and HLA-A02-negative analysis were identified as discoveries that could be attributed to HLA-A*02:01-specific mHAgs. The number of noncoding RAMAs was much larger than the number of coding RAMAs. Therefore, the threshold p-values for likelihood ratio tests and interaction tests used to identify noncoding discovery candidates were set at ≤10^-4^. Discovery RAMAs were tested in the CIBMTR replication cohort with Bonferroni adjustments for multiple comparisons. For Bonferroni adjustment, acute GVHD, chronic GVHD and relapse were treated as separate analyses, with grade 2-4, 2b-4, 3-4 acute GVHD and stage 2-4 gut GVHD considered as a single category. In addition, discoveries with expected hazard ratios >1.0 for acute and chronic GVHD and <1.0 for relapse were analyzed separately from discoveries with unexpected hazard ratios <1.0 for acute and chronic GVHD and >1.0 for relapse.

In a separate MHC-agnostic analysis, we screened for RAMAs independent of whether the donor and recipient pairs had any specific HLA allotype. For this purpose, the FHCRC cohort was randomized at a 3:2 ratio into discovery and replication cohorts with 1125 and 743 pairs, respectively. We initially identified coding RAMAs with likelihood ratio p-values <1.0 x 10^-3^ and noncoding RAMA with p-values <1.0 x 10^-5^ as discovery candidates. Because the number of candidates was too large to tolerate Bonferroni adjustment for multiple comparisons, we used p-values <1.0 x 10^-4^ for coding RAMAs and 1.0 x 10^-6^ for noncoding RAMAs as more stringent thresholds to prespecify discovery candidates for replication testing.

### Identification of Missense Proxies, Linkage Disequilibrium Groups, and Peptides Encoded by Variant Alleles, and Prediction of Processed Peptide Binding to HLA Allotypes

European ancestry linkage disequilibrium (LD) groups of genomic variants with pairwise r^2^ ≥0.70 were identified by the LDmatrix tool (ldlink.nci.nih.gov) (29). The LDproxy tool (ldlink.nci.nih.gov) was used to identify external missense proxy variants with r^2^ ≥0.70 in the European population to be included in linkage groups for analysis of HLA-A*02:01 RAMAs and to identify external proxy variants predicted to encode non-annotated open reading frames for analysis of MHC-agnostic RAMAs. When necessary, the LDproxy tool was also used to identify proxies with r^2^ ≥0.95 that could be used as backup assays for the primary variant being tested for replication in the CIBMTR cohort. Peptides up to 13 residues upstream and downstream from the amino acid encoded by genomic variants (i.e., source peptides) were recovered from Haplosaurus (ensembl.org) (30), the dbSNP database (ncbi.nlm.nih.gov/snp) (31) or from manual reading of the UCSC genome browser (genome-euro.ucsc.edu) (32). Some source peptides were shorter than 27 residues due to start or stop codons. The source peptides were then analyzed for predicted binding of 8 to 14-mer processed peptides to selected MHC class I allotypes by NetMHCpan4.1 at the default setting (33). The binding predictions are based on both the estimated binding affinity percentile rank and on publicly available data from mass spectrometry analysis of peptides eluted from HLA molecules. Processed peptides predicted to bind HLA-A*02:01 but not containing the variant residue were culled from further analysis.

## Results

**Figure 1:**
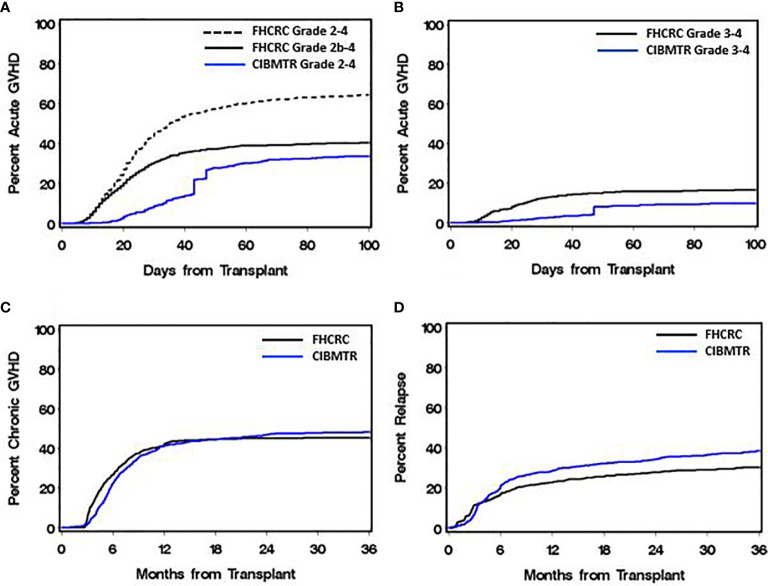
Cumulative incidence frequencies of acute GVHD, chronic GVHD and relapse between the discovery (FHCRC) and replication (CIBMTR) cohorts. **(A)** In the FHCRC cohort, grade 2-4 acute GVHD includes isolated stage 1 gastrointestinal GVHD, while grade 2b-4 acute GVHD excludes isolated stage 1 gastrointestinal GVHD. **(B)** Grade 3-4 acute GVHD. **(C)** Chronic GVHD was assessed by historical criteria in both cohorts. **(D)** Relapse includes recurrent or progressive malignancy after HCT.

**Table 1** summarizes demographic, clinical and transplant characteristics of patients in the study cohorts. Patients in the CIBMTR cohort were older than those in the FHCRC cohorts, fewer had chronic myeloid leukemia and low-risk diseases, and more received non-myeloablative conditioning regimens and growth factor-mobilized blood cell grafts. These differences reflect more recent HCT in the CIBMTR cohort than in the FHCRC cohorts. **Figure 1** shows the cumulative incidence frequencies of acute GVHD, chronic GVHD and relapse in the study cohorts. The cumulative incidence frequencies of grades 2-4 and grades 3-4 acute GVHD were higher in the FHCRC cohort (0.64 and 0.17, respectively) than in the CIBMTR cohort (0.36 and 0.10, respectively). The cumulative incidence frequencies of chronic GVHD and relapse were similar in the 2 cohorts (**Figure 1**).

**Table 1:** Characteristics of the study cohorts.

Characteristic, n (%)	FHCRC HLA-A*02:01-positive (n = 824)	FHCRC HLA-A02-negative (n = 929)	CIBMTR (n = 838)
Patient age at transplantation, y			
Median (range)	45 (0–72)	44 (0–74)	54 (2–74)
Diagnosis			
Acute leukemia	319 (39)	364 (39)	474 (57)
Chronic myeloid leukemia	176 (21)	226 (24)	30 (4)
Myelodysplastic syndrome or myeloproliferative neoplasm	130 (16)	139 (15)	201 (24)
Chronic lymphocytic leukemia	26 (3)	26 (3)	19 (2)
Malignant lymphoma or multiple myeloma	173 (21)	174 (19)	114 (14)
Disease risk*			
Low	177 (21)	215 (23)	19 (2)
Intermediate	204 (25)	251 (27)	443 (53)
High	397 (48)	412 (44)	357 (43)
Not classified	46 (6)	51 (5)	19 (2)
Donor-recipient gender			
Male to male	266 (32)	281 (30)	247 (29)
Male to female	174 (21)	190 (20)	197 (24)
Female to male	212 (26)	262 (28)	228 (27)
Female to female	172 (21)	196 (21)	166 (20)
Graft source			
Bone marrow	400 (49)	483 (52)	93 (11)
Mobilized blood cells	424 (51)	446 (48)	745 (89)
Conditioning			
Myeloablative < 900 cGy total body irradiation	389 (47)	417 (45)	428 (51)
Myeloablative ≥ 900 cGy total body irradiation	301 (37)	372 (40)	121 (14)
Nonmyeloablative	134 (16)	140 (15)	289 (34)

*Low risk is chronic myeloid leukemia in chronic phase or myelodysplastic syndrome-refractory anemia; intermediate risk, acute leukemia, chronic lymphocytic leukemia, or non-Hodgkin lymphoma in remission; high risk, all others.

### HLA-A*02:01-Positive Recipient Allele Mismatch Association Discoveries

**Figure 2:**
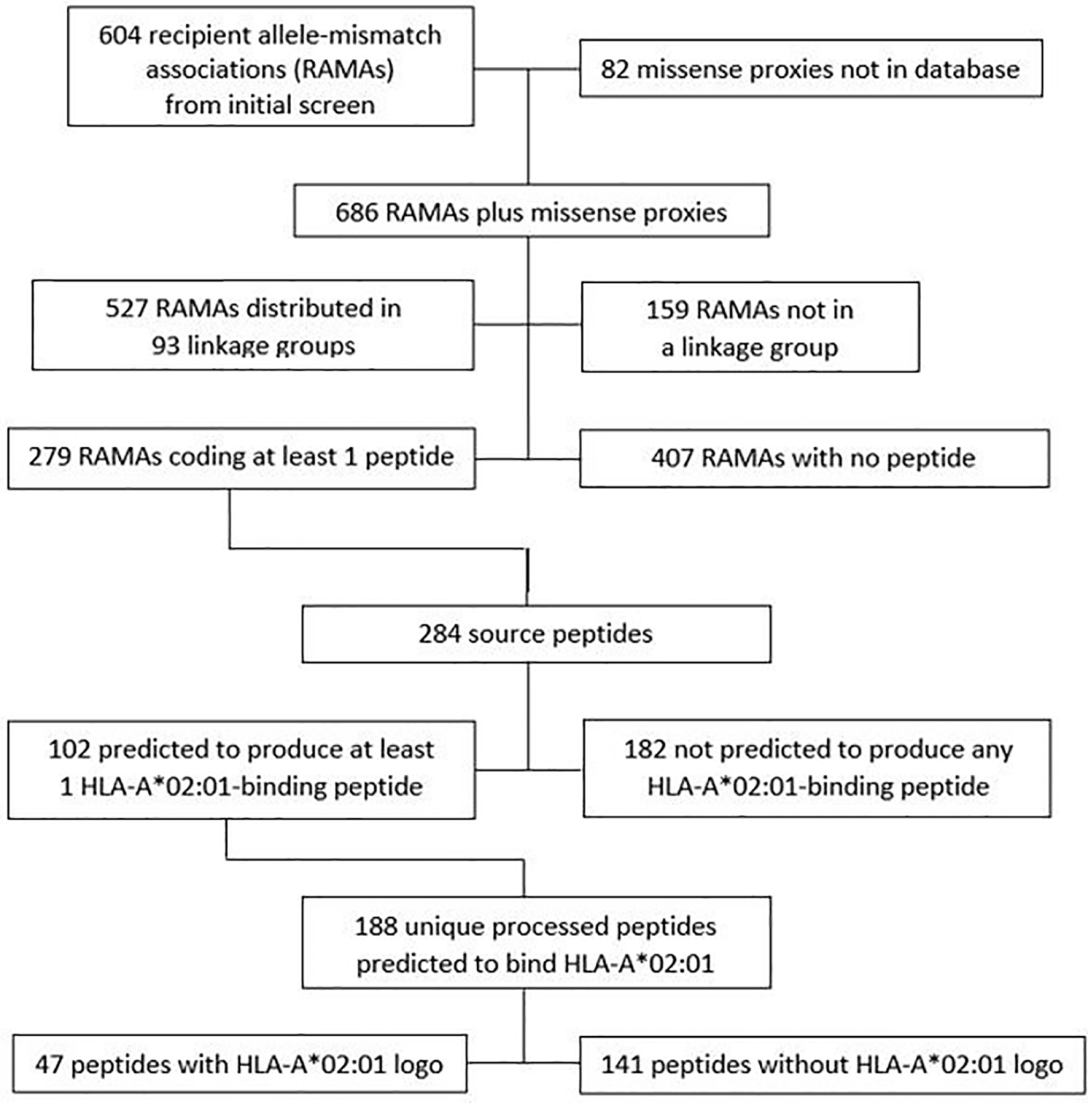
Discovery of recipient allele mismatch associations (RAMAs). Analysis of the discovery cohort identified 604 RAMAs. An additional 82 proxies were included because the variant encoded a missense peptide and is in LD (r^2^ ≥ 0.70) with a noncoding RAMA from the initial screen. 9-mer peptides with leucine, isoleucine, or methionine at the second position and valine, leucine, isoleucine, or methionine in the ninth position were characterized as having a sequence logo or pattern characteristic of peptides that bind to HLA-A*02:01.

The initial discovery analysis identified 604 variants associated with an outcome in the HLA-A*02:01-positive cohort and having a statistical interaction with the absence of HLA-A*02:01 in the combined HLA-A*02:01-positive and negative cohorts, indicating that these associations could be attributed to HLA-A*02:01-specific mHAgs (**Figure 2** and **Table S1**). Among the 604 discoveries, 197 were coding variants and 407 were non-coding variants. To characterize noncoding variants more accurately according to the presence or absence of any associated coding variants, we identified 82 external missense proxies with r^2^ ≥0.70 as additional RAMA discoveries. Of the 686 variants, 527 were combined into 93 linkage groups with pairwise r^2^ ≥0.70, and 159 did not have r^2^ ≥0.70 for association with any other variant in the data set. The 93 linkage groups contain between 2 and 105 members. Among the 686 RAMAs, 279 involved a variant that encodes at least 1 peptide, yielding a total of 284 unique source peptides (**Table S2**).

### Prediction of Peptides That Bind HLA-A*02:01

To validate the use of NetMHCpan4.1 (33) to identify source peptides that generate processed peptides predicted to bind to HLA-A*02:01, we tested its performance with a set of 49 previously identified genomic variants reported to encode mHAgs presented by HLA-A*02:01 (4, 8). The NetMHCpan4.1 algorithm predicted HLA-A*02:01 binding for all but 3 of the source peptides tested (**Table S3**). In 4 cases, the predicted peptide differed from the reported peptide, but the NetMHCpan4.1 algorithm predicted alternatives that approximated the reported peptide. Although this analysis could not determine the false-positive rate, the results show that the NetMHCpan4.1 algorithm has a low false-negative rate in predicting the binding of processed peptides to HLA-A*02:01.

**Figure 3:**
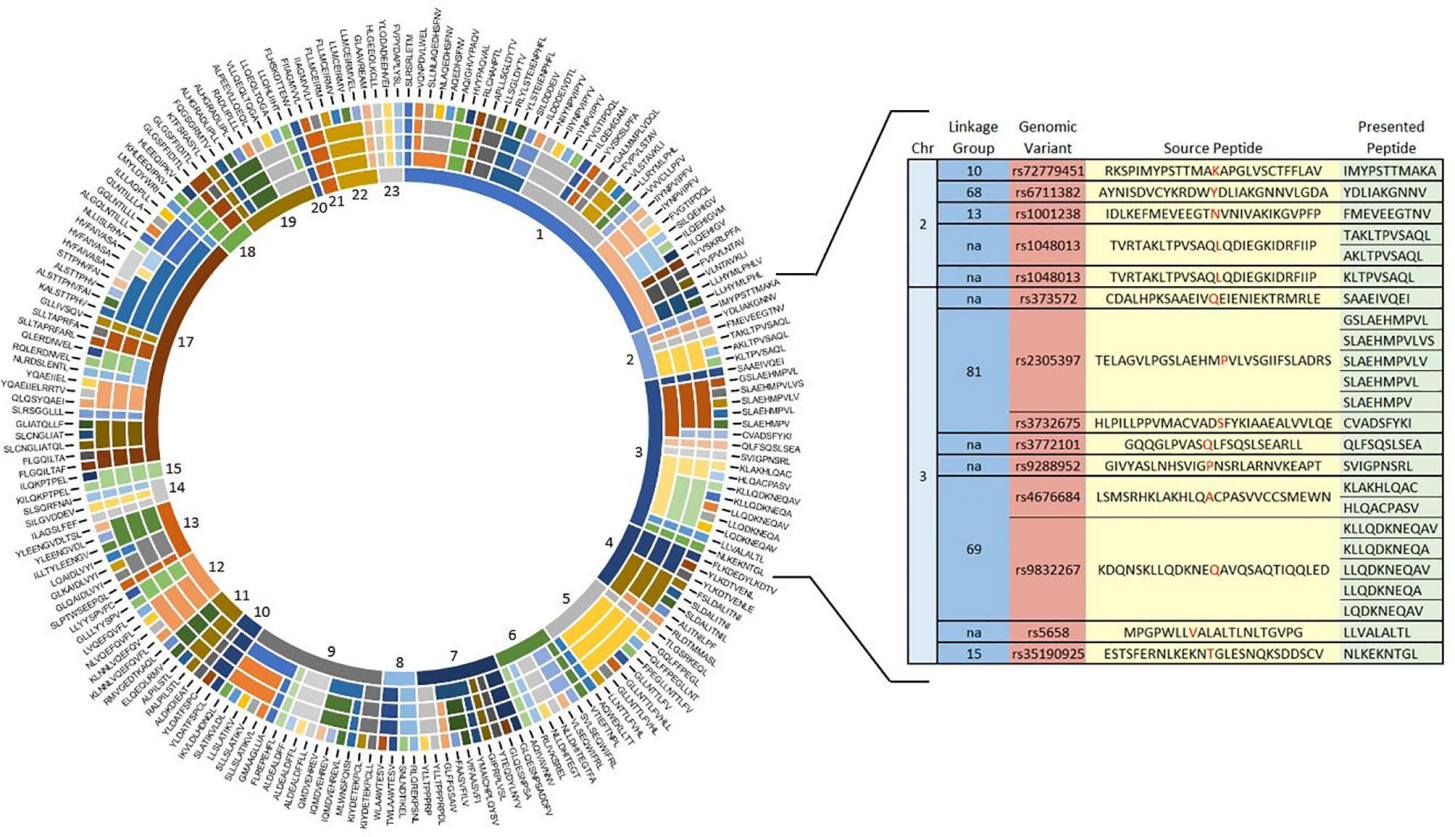
Outcomes associated with recipient mismatching for individual genomic variant discoveries may be explained by linkage with other variants collectively encoding sets of source peptides predicted to produce multiple processed peptides that bind HLA-A*02:01. From the innermost ring to the outermost ring, the doughnut chart shows numbered chromosomal locations, linkage groups, variant discoveries, source peptides and processed peptides predicted to bind HLA-A-*02:01. None of the discoveries was mapped to chromosome 16. See **Table S4** for source data. Details of discoveries in chromosomes (Chr) 2 and 3 show arbitrarily numbered linkage groups (dark blue column) of genomic variants (brown column) encoding source peptides (yellow column) predicted by NetMHCpan4.1 to produce processed peptides that bind HLA-A*02:01 (green column). Variant amino acids in the source peptides are identified in red font. Variants not included in a linkage group are designated “na.”.

Of the 284 unique source peptides identified in our discovery analysis, 102 have at least 1 processed peptide that i) contains 8 to 14 residues, ii) includes the amino acid encoded by the genomic variant, and iii) is predicted to bind to HLA-A*02:01 (**Figure 3** and **Table S4**). These 102 source peptides produce a total of 188 unique processed 8 to 14-mer peptides that are predicted to bind to HLA-A*02:01. Of these, 47 have a 9-mer HLA-A*02:01-binding pattern or sequence logo characterized by having leucine, isoleucine, or methionine at position 2 and valine, leucine, isoleucine, or methionine at position 9 (34, 35).

### Replication Testing of HLA-A*02:01 Discoveries

**Figure 4:**
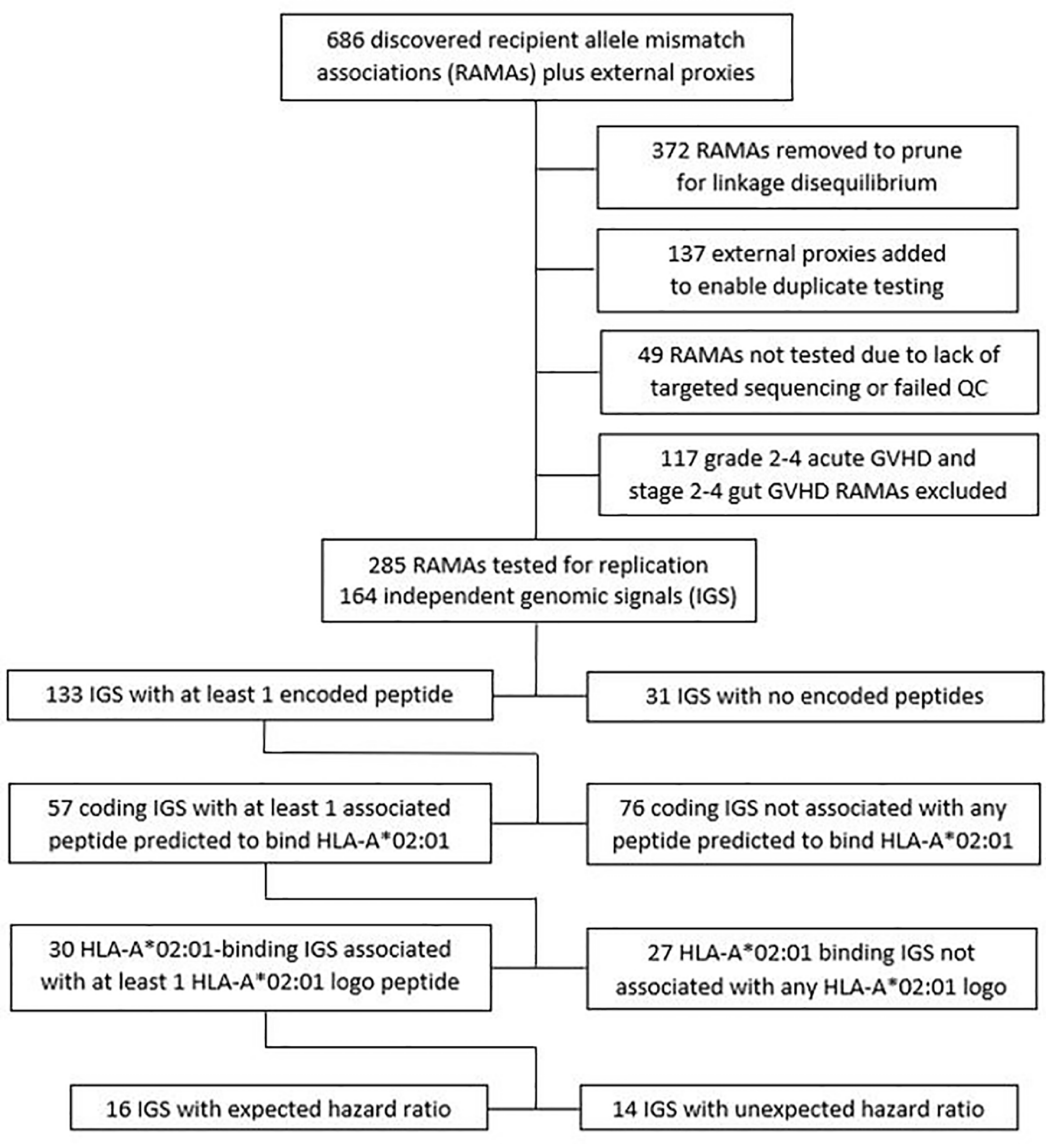
Replication testing of recipient allele mismatch associations (RAMAs) identified in the discovery cohort. Testing in the replication cohort was limited to no more than 2 members of each linkage group constituting an independent genomic signal (IGS). Discovery hazard ratios >1.0 for acute and chronic GVHD and <1.0 for relapse were categorized as expected, while discovery hazard ratios <1.0 for acute and chronic GVHD and >1.0 for relapse were categorized as unexpected. Each linkage group was characterized according to whether any variant in the group codes a source protein, is predicted to produce a processed peptide that binds to HLA-A*02:01 or to generate a peptide with an HLA-A*02:01 sequence logo.

For purposes of replication, 372 RAMAs were removed from the list of 686 discoveries and external proxies, so that no linkage group contained more than 2 variants, and 137 assay proxies with r^2^ >0.95 were added to enable backup testing of singleton RAMAs that did not fit within any linkage group. A total of 49 RAMAs were not evaluated due to lack of an appropriate assay or because the results did not pass QC after testing (**Figure 4** and **Table S5**). Grade 2b GVHD could not be ascertained in the CIBMTR cohort because gut staging was not available. Due to differences in the cumulative incidence frequencies of acute GVHD in the discovery and replication cohorts, we tested grades 2-4 GVHD in the CIBMTR cohort for replication of grades 2b-4 GVHD discoveries in the FHCRC cohort. We also tested replication for grades 3-4 GVHD, chronic GVHD and relapse, and excluded 117 RAMAs for grade 2-4 GVHD and stage 2-4 gut GVHD from replication testing.

Of the 285 RAMAs tested for replication, 121 represented backup assays within a linkage group, leaving 164 independent genomic signals (IGS). Among the 164 IGS, 83 (51%) had hazard ratios >1.0 as expected for acute and chronic GVHD or <1.0 as expected for relapse, while the remainder had unexpected hazard ratios <1.0 for acute or chronic GVHD or >1.0 for relapse. Each IGS was characterized according to whether any member of its linkage group encoded a source peptide, whether any processed peptides were predicted to bind to HLA-A*02:01, and whether any processed peptides had an HLA-A*02:01 sequence logo as defined above. Of the 164 IGS, 133 contained at least one coding RAMA. Among the 133, 57 contained at least one RAMA that was predicted to produce processed peptides that bind to HLA-A*02:01, and 30 of the 57 contained at least one RAMA that was predicted to produce a processed peptide with an HLA-A*02:01-binding sequence logo.

Among the 30 IGS associated with at least 1 HLA-A*02:01 sequence logo peptide, 16 had hazard ratios >1.0 as expected for acute and chronic GVHD and <1.0 as expected for relapse. These IGS with expected hazard ratios were prespecified to be evaluated formally for replication with statistical correction for multiple comparisons. These pre-specified IGS all had p-values >.05 for replication (**Table 2**). We also evaluated the 14 IGS that were associated with at least 1 HLA-A*02:01 sequence logo peptide but had unexpected hazard ratios. Likewise, these IGS all had p-values >.05 for replication (**Table 2**).

**Table 2:** HLA-A*02:01-restricted discoveries prespecified for replication testing.

SNP ID	Allele Tested	Phenotype	Discovery Results*					Replication Results†			
			HR	LB	UB	*P*	Exp	HR	LB	UB	*P*
rs2794751	C	agvhd2b-4	1.91	1.3	2.9	5.38E-03	Yes	1.06	0.6	1.8	0.83
rs2305398	A	agvhd2b-4	1.55	1.2	2.1	5.64E-03	Yes	1.18	0.8	1.6	0.34
rs5658	G	agvhd2b-4	1.79	1.2	2.7	7.28E-03	Yes	0.63	0.4	1.1	0.07
rs11937432	G	agvhd2b-4	2.10	1.4	3.2	2.01E-03	Yes	0.79	0.4	1.4	0.44
rs3816988	C	agvhd3-4	2.13	1.4	3.3	1.41E-03	Yes	1.37	0.7	2.5	0.33
rs698	T	cgvhd	1.74	1.2	2.4	2.89E-03	Yes	1.18	0.8	1.7	0.37
rs214950	A	cgvhd	1.52	1.1	2.0	4.83E-03	Yes	1.06	0.8	1.4	0.70
rs3735338	A	cgvhd	1.76	1.2	2.6	5.56E-03	Yes	1.14	0.8	1.5	0.42
rs4504745	G	relapse	0.55	0.3	0.9	8.38E-03	Yes	1.09	0.7	1.7	0.72
rs2290207	C	relapse	0.34	0.1	0.8	4.06E-03	Yes	0.94	0.5	1.6	0.83
rs9902235	C	relapse	0.29	0.1	0.8	2.23E-03	Yes	0.47	0.2	1.1	0.05
rs587404	A	relapse	0.53	0.4	0.8	5.45E-04	Yes	1.05	0.8	1.5	0.75
rs7536561	G	relapse	0.56	0.4	0.9	5.09E-03	Yes	0.74	0.5	1.1	0.13
rs1048013	C	relapse	0.56	0.4	0.8	3.08E-03	Yes	1.22	0.9	1.7	0.25
rs16869016	T	relapse	0.36	0.2	0.8	3.02E-03	Yes	0.81	0.5	1.3	0.38
rs17763658	A	relapse	0.28	0.1	0.7	5.23E-04	Yes	0.95	0.6	1.5	0.81
rs3856145	A	agvhd2b-4	0.50	0.3	0.8	3.41E-04	No	1.09	0.7	1.6	0.67
rs4676684	C	agvhd2b-4	0.61	0.4	0.9	4.64E-03	No	1.22	0.9	1.7	0.26
rs11544484	C	agvhd2b-4	0.32	0.1	0.9	6.07E-03	No	1.12	0.7	1.8	0.63
rs9051	C	agvhd2b-4	0.60	0.4	0.9	3.10E-03	No	0.85	0.6	1.2	0.40
rs685967	C	agvhd2b-4	0.32	0.1	0.8	1.81E-03	No	1.23	0.7	2.0	0.43
rs62622380	A	agvhd3-4	0.35	0.1	0.9	6.52E-03	No	0.53	0.2	1.4	0.17
rs7307331	A	agvhd3-4	0.35	0.2	0.7	1.29E-03	No	1.08	0.6	2.0	0.81
rs229526	C	agvhd3-4	0.35	0.1	0.8	5.88E-03	No	1.01	0.5	1.9	0.99
rs2955367	G	cgvhd	0.65	0.5	0.9	8.57E-03	No	0.74	0.5	1.0	0.06
rs3135507	T	cgvhd	0.39	0.2	0.8	3.38E-03	No	0.61	0.3	1.1	0.06
rs911973	A	cgvhd	0.36	0.2	0.8	3.21E-03	No	1.23	0.7	2.1	0.48
rs35190925	G	relapse	2.11	1.4	3.2	9.52E-04	No	1.06	0.7	1.6	0.80
rs2961144	A	relapse	2.06	1.4	3.1	2.18E-03	No	1.56	0.9	2.8	0.16
rs6259	A	relapse	1.91	1.3	2.8	1.61E-03	No	0.77	0.5	1.2	0.27

SNP ID, single nucleotide polymorphism identification; HR, hazard ratio; LB, lower boundary of the 95% confidence interval; UB, upper boundary of the 95% confidence interval; Exp, expected hazard ratio

*Discoveries represent results for FHCRC patients.

†Replication was tested in CIBMTR patients.

One IGS result that was not pre-specified drew scrutiny because the replication p-values for the primary and backup assays were 0.001 and 0.004, respectively. The replication hazard ratios for the association of recipient minor allele mismatching of rs62572859 and rs12554984 with the risk of grade 3-4 acute GVHD were 0.12 [95% confidence interval (CI), 0.02-0.84)] and 0.14 (95% CI, 0.02-0.99), respectively. In addition to the implausibility of the direction, the hazard ratios reflect a result based on an expectation of only 7 events in a few dozen patients (10% incidence of grade 3-4 acute GVHD, 9% probability of recipient allele mismatching for these variants, and ~800 genotyped pairs).

### Previously Identified HLA-A*02:01-Associated Minor Histocompatibility Antigens Are Not Associated With GVHD or Relapse After HCT

With the test for statistical interaction as a criterion, the 604 discovery RAMAs and 82 external missense proxies in **Table S1** included only 2 of the 49 previously identified variants that produce HLA-A*02:01-associated mHAgs (rs9051-G and rs9051-C) in **Table S3**. Very few of these 49 HLA-A*02:01-associated mHAgs have been tested for association with HCT outcomes. Therefore, we evaluated these alleles as candidates for association with all 6 HCT outcomes. For this analysis, the FHCRC HLA-A*02:01-positive cohort was randomized at a 3:2 ratio into discovery and replication cohorts with 486 and 338 donor and recipient pairs, respectively. Associations with p-values ≤.05 in the discovery cohort were tested in the replication cohort. The discovery analysis identified 17 RAMAs, 6 with expected hazard ratios and 11 with unexpected hazard ratios (**Tables S6**). These RAMAs all had p-values >.05 for replication (**Table 3**). **Table S7** summarizes result for the association of the 49 RAMAs with the 6 outcomes in the combined FHCRC discovery and replication cohorts to enable future meta-analysis with results from other cohorts.

**Table 3:** Known HLA-A*02:01 minor histocompatibility antigens prespecified for replication testing.

SNP ID	Allele Tested	Phenotype	Discovery Results					Replication Results			
			HR	LB	UB	*P*	Exp	HR	LB	UB	*P*
rs2273137	A	agvhd2-4	4.32	1.37	13.55	0.04	Yes	1.05	0.3	3.3	0.94
rs2273137	A	agvhd2b-4	5.78	1.83	18.26	0.02	Yes	*	*	*	0.09
rs892028	A	agvhd2b-4	5.27	1.62	17.21	0.03	Yes	2.99	0.7	12.7	0.20
rs299295	T	agvhd3-4	1.79	1.05	3.05	0.04	Yes	1.10	0.4	2.8	0.85
rs1805098	G	cgvhd	1.61	1.11	2.35	0.02	Yes	1.04	0.6	1.7	0.88
rs10004	A	relapse	*	*	*	0.01	Yes	0.56	0.2	1.8	0.28
rs2274217	T	agvhd2-4	0.52	0.33	0.81	0.002	No	1.20	0.8	1.8	0.37
rs11557236	A	agvhd2-4	0.56	0.31	1.03	0.04	No	1.95	1.0	4.0	0.10
rs9051	G	agvhd2-4	0.48	0.21	1.08	0.05	No	1.32	0.6	2.7	0.46
rs9051	G	agvhd2b-4	0.25	0.06	1.02	0.01	No	1.23	0.5	3.0	0.66
rs743582	G	agvhd2b-4	*	*	*	0.01	No	3.22	1.0	10.2	0.09
rs11556157	A	agvhd2b-4	0.28	0.07	1.11	0.02	No	0.86	0.3	2.7	0.80
rs2274217	T	agvhd3-4	0.30	0.10	0.96	0.01	No	1.46	0.7	3.1	0.35
rs27044	C	gut2-4	*	*	*	0.04	No	2.65	0.6	11.4	0.25
rs2274217	T	cgvhd	0.56	0.33	0.95	0.02	No	0.83	0.5	1.4	0.48
rs1138358	A	relapse	3.66	1.83	7.33	0.002	No	0.40	0.1	1.6	0.14
rs11136343	A	relapse	1.89	1.11	3.24	0.03	No	1.07	0.6	2.1	0.84

*A model could be fit but failed to converge due to lack of events, meaning HR = 0 but no valid standard error or confidence interval.

SNP ID, single nucleotide polymorphism identification; HR, hazard ratio; LB, lower boundary of the 95% confidence interval; UB, upper boundary of the 95% confidence interval; Exp, expected hazard ratio.

### HLA-A*02:01-Associated mHAg Peptides Bind to Other HLA-A and B Allotypes

**Figure 5:**
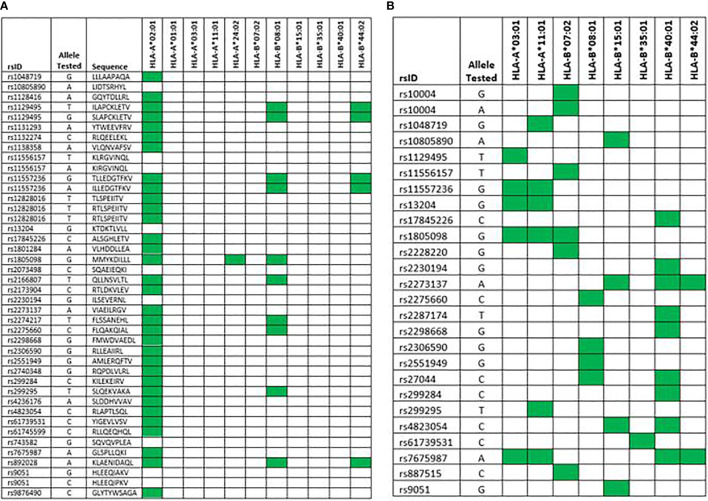
HLA-A*02:01-associated minor histocompatibility antigen peptides are predicted to bind promiscuously to other HLA-A and B allotypes. The HLA-allotypes shown in the columns include all that have allele frequencies >0.05 in the USA NMDP European Caucasian population. **(A)** Predictions are based on analysis of 9-mer and 10-mer peptides reported to bind HLA-A*02:01. Filled cells indicate the predicted binding of 9-mer and 10-mer peptides according to the PromPDD algorithm. **(B)** Predictions are based on analysis of HLA-A*02:01 minor histocompatibility antigen source peptides according to the NetMHCpan4.1 algorithm at the default setting. Filled cells indicate processed 9-mer peptides that include the variant amino acid residue and were identified as strong binders with <500 nM predicted affinity.

Our study was designed to identify mHAgs that can affect outcomes when presented by HLA-A*02:01 but not when presented by other HLA allotypes. Peptides that bind HLA class-1 molecules, however, show promiscuous binding to multiple allotypes both within and across supertypes (36–39). The extent to which the peptides presented by HLA-A*02:01 could bind to other HLA supertypes has not been determined. To address this question, we used the PromPDD algorithm (40) to evaluate the 42 9-mer and 10-mer peptides representing the 49 known HLA-A*02:01-associated mHAgs for binding to other HLA-A and B allotypes with allele frequencies >.05 in the USA NMDP European Caucasian population (A*01:01, A*03:01, A*11:01, A*24:02, B*07:02, B*08:01, B*15:01, B*35:01, B*40:01, B*44:02), none of which is included in the A02 supertype (41, 42). Approximately 80% of the individuals in this data set have at least 1 of the 4 HLA-A alleles or HLA-A*02:01, and approximately 66% have at least 1 of the 6 HLA-B alleles. As a positive control, the algorithm predicted that 34 (79%) of the 42 tested peptides bind to HLA-A*02:01 (**Figure 5A** and **Table S8**). Ten (24%) of the 42 peptides were predicted to bind to HLA allotypes outside the A02 supertype, including HLA-A*24:02 (n =1), HLA-*B08:01 alone (n = 4) and both HLA-B*08:01 and HLA*B44:02 (n = 5).

### HLA-A*02:01-Associated mHAg Source Peptides Produce Processed Peptides Predicted to Bind to Other HLA-A and B Allotypes

Minor histocompatibility antigens originate from source proteins or peptides that are processed through the MHC class I and class II antigen processing pathways for binding to MHC molecules (1, 2). To address the possibility that the source peptides that produce HLA-A*02:01-associated mHAgs could also produce processed peptides predicted to bind to other HLA allotypes, we used the NetMHCpan4.1 algorithm at the default setting to identify 9-mer peptides that include the variant amino acid residue and were identified as strong binders having a predicted affinity <500 nM for binding to the same HLA-allotypes listed above. Twenty-six (53%) of the 49 tested source peptides produced processed peptides that met these stringent specifications (**Figure 5B** and **Table S9**). Elution scores ranged from 0.33 to 0.96, indicating a high likelihood that these peptides have been detected in mass spectrometry studies. Binding score percentile ranks ranged from 0.01 to 1.03, indicating strong binding to the respective HLA allotype, and predicted binding affinities ranged from to 6.5 to 383 nM (**Table S9**). At least one processed 9-mer peptide was predicted to have strong binding to HLA-A*03:01 (n =5), HLA-A*11:01 (n = 6), HLA-B*07:02 (n = 6), HLA-B*08:01 (n = 4), HLA-B*15:01 (n = 4), HLA-B*35:01 (n = 1), HLA-B*44:02 (n = 9), and HLA-B*44:02 (n = 2) (**Figure 5B**). Four processed peptides were predicted to bind to 2 of the 10 tested HLA allotypes, 2 were predicted to bind to 3 of the 10 tested allotypes, and 1 was predicted to bind 4 of the 10 tested allotypes. No processed 9-mer peptides that include the variant amino acid were predicted to have strong binding to HLA-A*01:01 or HLA-A*24:02.

### Discovery and Replication of MHC-Agnostic RAMAs

The test for statistical interaction with HLA-A*02:01 as a criterion for discovery eliminated many possible mHAgs from consideration. To address this limitation, we used an MHC-agnostic approach to identify mHAgs associated with outcomes in the FHCRC cohort (10). The initial screen identified 802 variants, 403 with expected hazard ratios and 399 with unexpected hazard ratios (**Table 4** and **Table S10**). Pruning for linkage disequilibrium (LD) yielded 285 IGS, 142 with expected hazard ratios and 143 with unexpected hazard ratios. By using 10-fold smaller p-value thresholds, we prespecified 28 signals to be tested for replication in categories defined according to whether the discovery hazard ratio was expected or not and whether the IGS included coding variants or not. None of the 17 prespecified signals with unexpected hazard ratios met criteria for replication (**Table S11**). Of the 11 prespecified signals with expected hazard ratios, 1 signal met criteria for replication with Bonferroni adjustment for multiple comparisons, and 1 other signal came close to meeting criteria (**Table S11**). **Table S12** summarizes result for the association of the 802 MHC-agnostic RAMAs with the 6 outcomes in the combined FH discovery and replication cohorts to enable future metanalysis with results from other cohorts.

**Table 4:** Discovery and replication of MHC-agnostic recipient allele mismatch associations.

Variant Category‡	Discoveries*			Replication†	
	Variants	Signal-phenotypes	Signals^‖^	Prespecified Signals	Replicated Signals
Expected hazard ratio	403	150	142		
Acute GVHD	348	111	103	10	1
Grade 2-4	57	35			
Grade 2b-4	164	44			
Grade 3-4	77	22			
Stage 2-4 gut	50	10			
Chronic GVHD	35	24	24	1	0
Relapse	20	15	15	0	
Unexpected hazard ratio	399	148	143		
Acute GVHD	264	91	86	11	0
Grade 2-4	97	35			
Grade 2b-4	64	26			
Grade 3-4	52	21			
Stage 2-4 Gut	51	9			
Chronic GVHD	35	23	23	1	0
Relapse	100	34	34	5	0

*Discovery candidates included all coding variants with p-values <1.0 x 10^-3^ and noncoding variants with p-values <1.0 x 10^-5^.

†Replication tests were limited to coding variants with p-values <1.0 x 10^-4^ and noncoding variants with p-values < 1.0 x 10^-6^.

‡Expected hazard ratios (HR) were >1.0 for acute and chronic GVHD and <1.0 for relapse. Unexpected HRs were <1.0 for acute and chronic GVHD and >1.0 for relapse.

^‖^Small numbers of signals were associated with more than one subcategory of acute GVHD.

**Figure 6:**
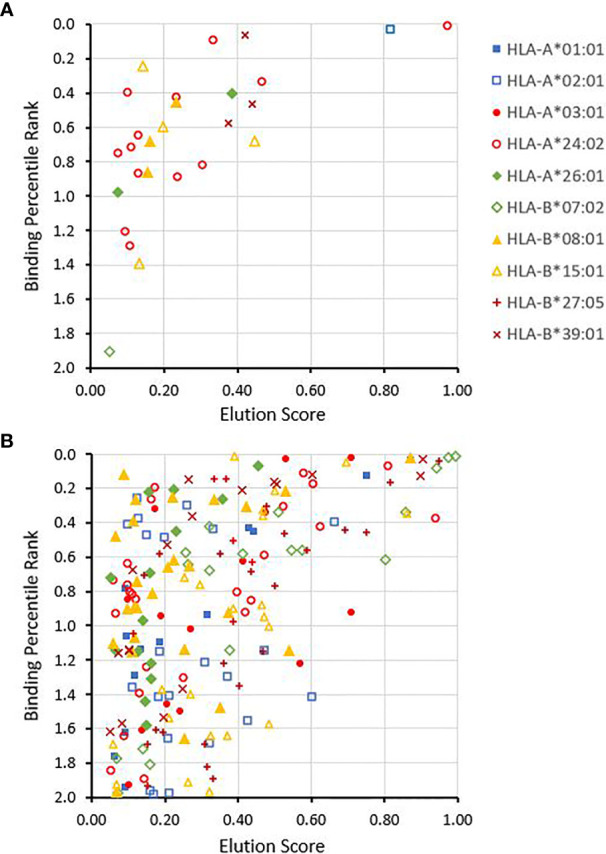
Annotated noncoding regions containing recipient allele mismatch associations have open reading frames producing processed 8-11mer processed peptides that contain the variant amino acid residue and are predicted to have promiscuous binding to a variety of HLA-A and B allotypes. Peptides originate from variants associated with **(A)** rs56040842 and **(B)** rs17811627. Peptides with elution scores <0.05 or percentile binding ranks >2.0 were excluded.

The prespecified signal that met criteria for replication contained 2 variants, rs56040842 and rs61851500. The replication hazard ratios for the association of recipient minor allele mismatching of rs56040842 and rs61851500 with the risk of grade 2b-4 acute GVHD were 3.38 (95% CI, 1.9-6.1; p = .0006) and 2.71 (95% CI, 1.5-4.8; p = .004), respectively. These hazard ratios reflect results based on an expectation of only 9 events in a score of patients (40% incidence of grade 2b-4 acute GVHD, 3% probability of recipient allele mismatching for these variants, and ~750 genotyped pairs). According to LDproxy, the signal defined by recipient minor allele mismatching of rs56040842 and rs61851500 contains only 1 other variant in LD with pairwise r^2^ ≥ 0.7 (rs61851533), and all 3 variants are in a *PRKG1* intron. Because intron sequences can encode non-annotated proteins (43–45), we used the ExPASy Translate tool (web.expasy.org/translate) to identify open reading frames that include the variant allele and encode an amino acid sequence that differs from the major allele. With this process we identified 4 such open reading frames (**Table S13**), none of which map to an annotated coding region as determined by the BLATP tool (ensembl.org). We used NetMHCpan4.1 to determine whether any of the potential source peptides are predicted to produce processed 8-11mer peptides with elution scores >0.05 and predicted percentile ranks ≤2.0 for binding to highly prevalent HLA-A and B allotypes as described above. This screening identified 27 peptides that are collectively predicted to bind to a range of HLA-A and B allotypes, including HLA-A*02:01, HLA-A24:02, HLA-A*26:01, HLA-B*07:02, HLA-B*08:01, HLA-B*15:01, HLA-B*39:01 and HLA-B*58:01 (**Figure 6A** and **Table S14**).

One other variant that came close to meeting criteria for replication was rs17811627. The signal containing this variant included 4 other variants in the database, all having pairwise r^2^ >0.82 with the others. For rs17811627, the replication p-value for the association of mismatching for the minor allele with grade 3-4 GVHD was 0.013, whereas the Bonferroni adjusted threshold was 0.125. Replication p-values for the other 4 variants in the signal ranged from.02 to.04. The replication hazard ratio for rs17811627 was 1.84 (95% CI, 1.2-2.9).

According to LDproxy, the signal containing rs17811627 includes 38 variants having pairwise r^2^ ≥0.70 with the others, all located in an intergenic region spanning 46 Kb on chromosome 15. Because intergenic regions can encode non-annotated proteins (45), we used the process described above to identify 30 open reading frames that include the minor allele and encode an amino acid sequence that differs from the major allele (**Table S15**), none of which map to an annotated coding region as determined by the BLATP tool. NetMHCpan4.1 screening identified a total of 151 8-11mer peptides each with predicted binding to at least 1 of the 12 HLA-A and B allotypes tested (**Figure 6B** and **Table S16**). Each tested HLA-allotype was predicted to bind between 13 and 28 peptides, and 11 of the 151 peptides were predicted to bind between 3 and 5 of the HLA-allotypes tested.

## Discussion

Our initial approach toward identification of mHAgs associated with outcomes after HCT was based on the simplifying assumption that mHAgs presented by HLA-A*02:01 could be efficiently identified by their association with clinical outcomes in recipients with at least 1 HLA-A*02:01 allele but not in other recipients. Our results show that this assumption is belied by LD and by promiscuous binding of HLA-A*02:01 peptide ligands to other HLA allotypes within and across HLA-A and B supertypes. We used rigorous peptide binding predictions to prespecify the most plausible discovery associations to test for replication. Even so, with 1 possible exception, none of the HLA-A*02:01 discovery associations was replicated in an independent cohort, whether prespecified or not.

We cannot exclude the possibility that differences between the FHCRC cohort and the CIBMTR cohort contribute to the lack of replication. On the other hand, previous studies have not identified clinical variables that interact with the association between recipient HLA-mismatching and the risks of GVHD. Regardless of the underlying disease, year of transplant, intensity of the conditioning regimen and other clinical variables, recipient HLA-mismatching is consistently associated with an increased risk of GVHD. Therefore, we have no reason to expect that clinical variables interact with the effects of recipient mHAg mismatching.

Other factors more likely explain our inability to identify RAMAs with acute GVHD, chronic GVHD or relapse after allogeneic HCT. First, the effect size of any GVHD associations is not likely to exceed 2.0. In one large study, the hazard ratios for the association of recipient HLA mismatching with grade 2-4 acute GVHD and chronic GVHD after related HCT at our center were 1.7 (95% CI, 1.49-2.03) and 1.24 (95% CI, 0.95-1.60), respectively (46). The increased risk of GVHD can be explained by the cumulative effect of T cell responses against recipient-specific HLA-epitopes and the distinct repertoires of peptides presented by the mismatched HLA allotypes in the recipient. Hence, the incremental effect of any given recipient mHAg mismatch must be smaller than the effect of an HLA mismatch. Second, the probability of recipient allele mismatching averages around 10% and is combined with event frequencies of 10-40%. With such small sample sizes, confidence intervals are wide. Third, although in silico tools have some ability to predict peptide binding to specific HLA-allotypes, binding per se does not necessarily predict an immune response or an altered clinical outcome even if an immune response were to occur. Whether the risks of acute and chronic GVHD and relapse reflect a few mismatches that have large effects or many mismatches each having a small effect is not known, although our results suggest that the latter may be more plausible than the former, making it unlikely that mHAg matching could be used for donor selection or risk stratification.

We did not expect to find a high proportion of discovery RAMAs with hazard ratios <1.0 for acute or chronic GVHD or >1.0 for relapse. No mechanism is available to explain how an MHC class-1-presented mHAg could prevent immune responses against a wide variety of other class 1-presented mHAgs. It is conceivable that members of a linkage group tagged by a genomic variant could encode class II mHAgs that selectively trigger Treg cells. If so, the clinical outcomes could depend on the overall balance between activation and regulation of alloactivated donor T cells, but we have no evidence to support this speculation.

Beyond the unexpected hazard ratio, the HLA-A*02:01 results for rs62572859 and rs12554984 are difficult to explain. Although the minor allele of rs62572859 encodes a peptide, it is not predicted to bind HLA-A*02:01, and rs12554984 is an intron variant with no open reading frames that include the variant allele. Forty-five other variants have pairwise r^2^ values >0.70 for association with rs62572859 in Europeans, but none of these is a coding variant. We cannot exclude the possibility that this association is explained by non-annotated open reading frames but given the unexpected hazard ratio and the low numbers of informative patients, we caution that this result should be considered as a statistical outlier until proven otherwise.

Our MHC-agnostic analysis does not allow any definitive conclusions regarding the association of individual genomic variants with the risk of acute GVHD. Results for the signal tagged by rs56040842 are based on low numbers of informative patients, raising concern that it represents a statistical outlier, even though this association was replicated in an independent cohort. *PRKG1* protein is expressed in the skin, stomach and colon, tissues that are targets of acute GVHD. Results of the NetMHCpan4.1 analysis suggest that peptides corresponding to intronic open reading frames are associated with a variety of HLA-A and B allotypes, and the BLATP results exclude the possibility that these peptides can be attributed to annotated proteins. Results for the signal tagged by rs17811627 are based on larger numbers of informative patients, but the p-values did not meet the prespecified Bonferroni-adjusted threshold of statistical significance. The predicted HLA-A and B-binding peptides cannot be attributed to annotated proteins, but no evidence is available to demonstrate that any non-annotated open reading frames encoded within the intergenic region tagged by rs17811627 are expressed in GVHD target tissues.

Our results beg the question of how mHAgs should be defined. Under *in vitro* conditions, an individual mHAg can be identified as a complex composed of a defined-peptide and a defined-MHC allotype, which evokes an immune response. Efforts are in progress to determine whether T cells that recognize selected mHAgs could be used to eliminate malignant cells in HCT recipients (47–52). From the perspective of allogeneic HCT between a pair of HLA-identical siblings, a mHAg manifests as a set of peptides originating from annotated proteins and non-annotated open reading frames, which i) are encoded by a group of recipient genomic mismatches in high LD, ii) bind to HLA allotypes in the recipient, and iii) evoke a donor immune response. Attribution of the immune response and clinical outcomes to individual components within a peptide x HLA allotype matrix as large and complex as the one tagged by rs17811627 would require extensive *in vitro* studies, and the results would likely differ from patient to patient according to their HLA types. Our results also support the possibility that peptides from annotated proteins and non-annotated open reading frames (44, 45, 53, 54) both contribute to immune-mediated outcomes after HCT between HLA-identical siblings.

## Data Availability Statement

The datasets presented in this study can be found in online repositories. The names of the repository/repositories and accession number(s) can be found below: BioSample, phs001918.

## Ethics Statement

The studies involving human participants were reviewed and approved by Fred Hutchinson Cancer Research Center. Written informed consent to participate in this study was provided by the participants or their legal guardian/next of kin.

## Author Contributions

JH, PM, DL, and BS developed the study concept and design. DL and BS managed data acquisition, variant imputation, informatics analyses, quality control and statistical analyses. DL and XZ provided HLA imputation and genomic mismatch calculations. DJ and BH provided a database of variant annotations. BN and DG assisted with identification of source proteins. SS provided samples for the CIBMTR cohort. CS and FW provided genotyping of CIBMTR samples. PM, DL, and BS interpreted results and wrote the manuscript. All authors other than JH revised draft manuscripts and approved the final version of the manuscript. JH died on July 31, 2019.

## Funding

This work was supported by grants from the National Institutes of Health AI33484, AI049213, CA015704, CA18029, HL087690, HL088201, HL094260 and HL105914. Deidentified samples and data were provided by the CIBMTR supported by primarily by Public Health Service U24CA076518 from the National Cancer Institute (NCI), the National Heart, Lung and Blood Institute (NHLBI) and the National Institute of Allergy and Infectious Diseases (NIAID), by HHSH250201700006C from the Health Resources and Services Administration (HRSA), and by N00014-20-1-2705 and N00014-20-1-2832 from the Office of Naval Research. Genotyping of these samples was done by the Genomics shared resource of the Fred Hutch/University of Washington Cancer Consortium supported by the National Cancer Institute (P30 CA015704).

## Conflict of Interest

The authors declare that the research was conducted in the absence of any commercial or financial relationships that could be construed as a potential conflict of interest.

## Publisher’s Note

All claims expressed in this article are solely those of the authors and do not necessarily represent those of their affiliated organizations, or those of the publisher, the editors and the reviewers. Any product that may be evaluated in this article, or claim that may be made by its manufacturer, is not guaranteed or endorsed by the publisher.
